# LettuceDB: an integrated multi-omics database for cultivated lettuce

**DOI:** 10.1093/database/baae018

**Published:** 2024-04-01

**Authors:** Wenhui Zhou, Tao Yang, Liucui Zeng, Jing Chen, Yayu Wang, Xing Guo, Lijin You, Yiqun Liu, Wensi Du, Fan Yang, Cong Hua, Jia Cai, Theo van Hintum, Huan Liu, Ying Gu, Xiaofeng Wei, Tong Wei

**Affiliations:** College of Life Sciences, University of Chinese Academy of Sciences, Beijing 100049, China; BGI Research, Wuhan 430074, China; State Key Laboratory of Agricultural Genomics, Key Laboratory of Genomics, Ministry of Agriculture, BGI Research, Shenzhen 518083, China; China National GeneBank, BGI Research, Shenzhen 518120, China; Guangdong Genomics Data Center, BGl research, Shenzhen 518120, China; BGI Research, Wuhan 430074, China; South China Agricultural University, Guangzhou 510642, China; China National GeneBank, BGI Research, Shenzhen 518120, China; State Key Laboratory of Agricultural Genomics, Key Laboratory of Genomics, Ministry of Agriculture, BGI Research, Shenzhen 518083, China; BGI Research, Wuhan 430074, China; State Key Laboratory of Agricultural Genomics, Key Laboratory of Genomics, Ministry of Agriculture, BGI Research, Shenzhen 518083, China; China National GeneBank, BGI Research, Shenzhen 518120, China; China National GeneBank, BGI Research, Shenzhen 518120, China; China National GeneBank, BGI Research, Shenzhen 518120, China; China National GeneBank, BGI Research, Shenzhen 518120, China; BGI Research, Wuhan 430074, China; China National GeneBank, BGI Research, Shenzhen 518120, China; BGI Research, Wuhan 430074, China; China National GeneBank, BGI Research, Shenzhen 518120, China; Centre for Genetic Resources, the Netherlands, P.O. Box 16, Wageningen 6700 AA, The Netherlands; State Key Laboratory of Agricultural Genomics, Key Laboratory of Genomics, Ministry of Agriculture, BGI Research, Shenzhen 518083, China; BGI Research, Wuhan 430074, China; State Key Laboratory of Agricultural Genomics, Key Laboratory of Genomics, Ministry of Agriculture, BGI Research, Shenzhen 518083, China; China National GeneBank, BGI Research, Shenzhen 518120, China; Guangdong Genomics Data Center, BGl research, Shenzhen 518120, China; BGI Research, Wuhan 430074, China; State Key Laboratory of Agricultural Genomics, Key Laboratory of Genomics, Ministry of Agriculture, BGI Research, Shenzhen 518083, China

## Abstract

Crop genomics has advanced rapidly during the past decade, which generated a great abundance of omics data from multi-omics studies. How to utilize the accumulating data becomes a critical and urgent demand in crop science. As an attempt to integrate multi-omics data, we developed a database, LettuceDB (https://db.cngb.org/lettuce/), aiming to assemble multidimensional data for cultivated and wild lettuce germplasm. The database includes genome, variome, phenome, microbiome and spatial transcriptome. By integrating user-friendly bioinformatics tools, LettuceDB will serve as a one-stop platform for lettuce research and breeding in the future.

**Database URL**: https://db.cngb.org/lettuce/

## Introduction

Omics encompasses disciplines such as genomics, transcriptomics, proteomics, metabolomics and phenomics (1, 2). Focusing solely on individual data types leads to a limited and incomplete understanding biological process, while integrating multiple omics datasets enables a more comprehensive interpretation (3, 4). With advancing sequencing technologies and bioinformatics tools, extensive multi-omics studies have been conducted on various crop species and abundant omics data have been generated in the last decade (5–7). The first steps to break the barriers of multi-omics data is to integrate such data and promote data sharing through public databases. For example, the recent SoyOmics, Brassica napus multi-omics infotmation resource and ZEAMAP have integrated multi-omics data along with germplasm information, which facilitate data sharing and utilization for soybean, rapeseed and maize (8–10). These databases cover diverse data types, including genome, variome, transcriptome and phenome, and offer various tools to visualize and retrieve the omics data (8–10).

Cultivated lettuce (*Lactuca sativa*) is a nutritious vegetable crop and cultivated worldwide as an important agricultural commodity (11, 12). The reference genome was published in 2017 (13), based on which the Lettuce Genome Resource website (https://lgr.genomecenter.ucdavis.edu/) was established as a portal for lettuce genetic and genomic resources. Several multi-omics studies have been conducted since then and provided valuable resources for functional study and molecular breeding. A population transcriptome study of 240 cultivated and wild lettuce accessions revealed the single domestication event for cultivated lettuce and identified a regulatory network for anthocyanin biosynthesis (14). In 2021, a more comprehensive population genomics study of 445 *Lactuca* accessions revealed the domestication history of cultivated lettuce and identified genomic regions associated with important agronomic traits (15). Therefore, an integrative database is needed for ever-accumulating omics data, especially from multidisciplinary approaches.

**Figure 1.**
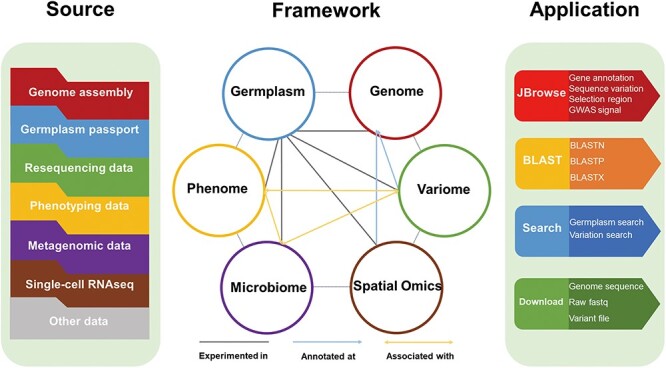
The framework of LettuceDB, including six data modules and bioinformatics tools.

Here in this study, we developed LettuceDB (https://db.cngb.org/lettuce/), a multi-omics database for lettuce research and breeding (Figure 1). LettuceDB integrated multi-omics data, including genome, variome, phenome, microbiome and spatial transcriptome from 445 *Lactuca* accessions worldwide. Additionally, gene annotations and association signals were provided in a user-friendly JBrowse interface, and a bioinformatics toolkit was deployed for laboratory users. LettuceDB provides an integrative platform of germplasm information and multi-omics data for cultivated lettuce, which facilitates a better access to the public data and utilization in research and breeding.

## Material and methods

### Data sources

#### Genomic data

Version 8 (v8) of genome assembly and its annotation were downloaded from CoGe (https://genomevolution.org/coge//GenomeInfo.pl?gid=28333) (13); the genome assembly and annotation of Version 11 (v11) were downloaded from National Center for Biotechnology Information (https://ftp.ncbi.nlm.nih.gov/genomes/all/GCA/002/870/075/GCA_002870075.4_Lsat_Salinas_v11). The relative proportion of the nucleotides Guanine (G) and Cytosine (C) in the genome was calculated within a window size of 100 kb and a step of 100 kb using a custom python script.

#### Germplasm and phenome data

The germplasm and phenotypic records of the 445 lettuce accessions were retrieved from the website of the Center for Genetic Resources, the Netherlands (https://www.wur.nl/en/research-results/statutory-research-tasks/centre-for-genetic-resources-the-netherlands/plant-genetic-resources/genebank/cgn-crop-collections/cgn-leafy-vegetables-collection/cgn-lettuce-collection.htm).

#### Variome, microbiome and spatio-temporal transcriptome data

Raw sequencing data were obtained from the previously published work (15) and used for variant calling based on the Version 11 genome assembly with the Genome Analysis Toolkit (Version 4.4.0.0). Briefly, raw reads were filtered using Trimmomatic (Version 0.39) (16), followed by bwa alignment and polymerase chain reaction duplicate identification. The variant calling step was conducted using a bioinformatics analysis accelerator MegaBOLT (17) module in the Genomic Variant Call Format mode, and single nucleotide polymorphisms (SNPs) and indels were identified using the joint calling approach (18). Population analysis and genome-wide association study (GWAS) analysis were conducted as in the previous study using Efficient Mixed-Model Association eXpedited (Version beta-07Mar2010) (15). For microbiome, rhizosphere soil samples from 2-month Salinas plants were subjected for microbial DNA extraction, and 16S rRNA gene was amplified for sequencing in a DIPSEQ\ platform as previously described (15), from which operational taxonomic units were identified as previously described (19). For spatial transcriptome, 2-month Salinas leaf was harvested for single-cell RNA sequencing.

### Haplotype analysis

A haplotype network analysis of the *KN1* gene and 2-kb flanking sequences was constructed to determine the genealogical relationship using PopArt (Version 1.7) (20). A simple (*ε* = 0) median-joining network was computed using SNPs with a missing rate of <10% and a minor allele frequency (MAF) of >0.05.

### Implementation

We constructed the frontend framework using Vue.js (Version 2.6.14) and built the backend using Django (Version 2.2) and Python (Version 3.7.4). LettuceDB used PostgreSQL (Version 9.6) to store the metadata of germplasm, genome, variome, phenome, microbiome and STOmics. We used Elasticsearch (Version 7.16.2) as the search engine in the resource center of LettuceDB. To manage and visualize curated datasets, we employed MongoDB (Version 4.2) and JBrowse (Version 2.0). We used Redis (v5.0.4) as the cache to store and manage the data in memory. For task queue management, we applied RabbitMQ (v3.8.13). Nginx (v1.20.1) was used as the reverse proxy server. Currently, LettuceDB supports the following browsers: Google Chrome (v80.0 and above), Opera (v62.0 and above), Safari (v12.0 and above) and Firefox (v80.0 and above).

## Results

### Reanalysis of the resequencing data using a new reference genome of cultivated lettuce

**Figure 2.**
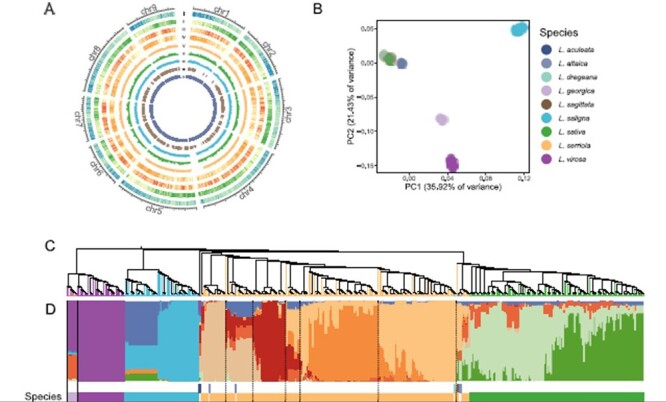
Variant calling and population structure of the sequenced *Lactuca* accessions. (A) The distribution of genomic features and variant density based on the new v11 genome assembly. The Circos plot shows from the outmost gene density (I), GC content (II), SNP count (III), indel count (IV), nucleotide diversity in *L. serriola* (V), nucleotide diversity in *L. sativa* (VI), fixation index between *L. serriola* and *L. sativa* (VII) calculated in 1-Mb sliding windows and selective sweeps (VIII) and newly assembled region in the v11 assembly (IX). (B) PCA of 440 *Lactuca* accessions. (C) A neighbor-joining tree. (D) Model-based clustering analysis with ancestry kinship of 10.

Variant identification from resequencing data relies on a high-quality reference genome. Since the release of the variant files from our previous study (15), a new version of lettuce reference genome was released to public. New Version 11 was derived from the same cultivar, Salinas, as the previous v8 assembly, which had a higher quality in terms of ungapped length (2.6 Gb in v11 and 2.2 Gb in v8), scaffold numbers (91 in v11 and 9958 in v8) and scaffold N50 (324.7 Mb in v11 and 1.8 Mb in v8). The comparison between two assemblies revealed a total of 374.8 Mb newly anchored sequences in the v11 assembly (Figure 2A). Because this assembly represents a major update in the genome continuity and quality, in this study, we reanalyzed the resequencing data using this v11 assembly.

A total of 228 138 802 SNPs and 32 224 494 indels were identified based on the v11 assembly (Figure 2A). This dataset had 27.6% more of SNPs and 9.1% more of indels than the previous study (15), which reflects the necessity of variant calling using the new assembly. The population analyses were rerun on the new SNP set after filtering with a missing rate of <10% and a MAF of >0.05. Principal component analysis (PCA) and admixture revealed the same population structure as previously reported (15), and the association analyses of eight agronomic traits and 22 *Bremia* resistance trials identified the similar genomic regions (Figure 2B–D). However, cross-population composite likelihood ratio test (XP-CLR) using the newly identified SNPs identified 4850 selective sweeps affecting a total of 156.8 Mb and 4345 genes (Circle VIII in Figure 2). Compared with the previously reported 107.7 Mb, we identified additional 49.1-Mb regions under selection with 11.6 Mb from the newly assembled region.

Using the newly released reference genome, we identified more than 52 million new variants compared with the present dataset and revealed additional selective sweeps. We therefore developed an updated lettuce database based on this new variant set along with the previous one.

### Overview of LettuceDB

**Figure 3.**
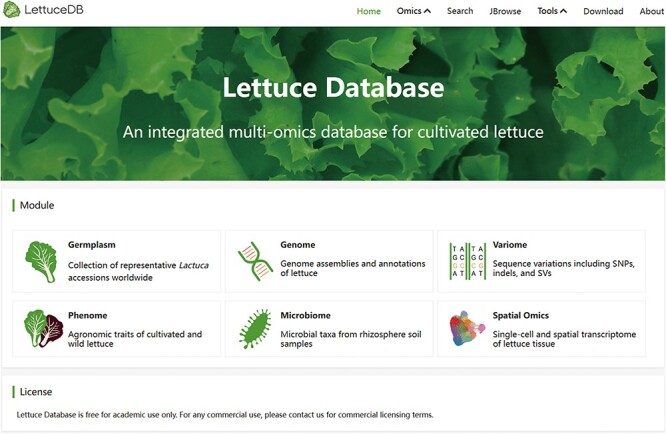
The LettuceDB home page.

To build a comprehensive multi-omics database, we collected multi-omics data and developed into six interactive modules: Gemplasm, Genome, Variome, Phenome, Microbiome and STOmics. The Germplasm module includes the passport information of 445 *Lactuca* accessions (Figure 3). The Genome module provides genome sequences and annotations from two versions of assemblies. The Variome module provides sequence variations based on two versions of lettuce reference genomes from the 445 accessions. The Phenome module provides 54 agronomic traits provided by the Centre for Genetic Resources, the Netherlands. The Microbiome module provides the abundance of representative microbial species in the rhizosphere samples from different *Lactuca* accessions. The STOmics provides single-cell data of cultivated lettuce.

LettuceDB provides a user-friendly interface with interactive plots and searching bars in each module. The powerful search engine presents comprehensive results with friendly links between omics modules for seamless navigation. LettuceDB also integrates multi-omics data into the genome browser, JBrowse, that provides reference sequence, annotation, population statistics and genome-wide-associated analysis results for agronomic traits. The tool page offers a plethora of bioinformatics tools, including Basic Local Alignment Search Tool (BLAST), LiftOver, Selective test and GWAS Single-Trait for laboratory research. A detailed instruction is provided in the About page. Therefore, LettuceDB provides an integrative platform for lettuce research and breeding.

### The germplasm module

**Figure 4.**
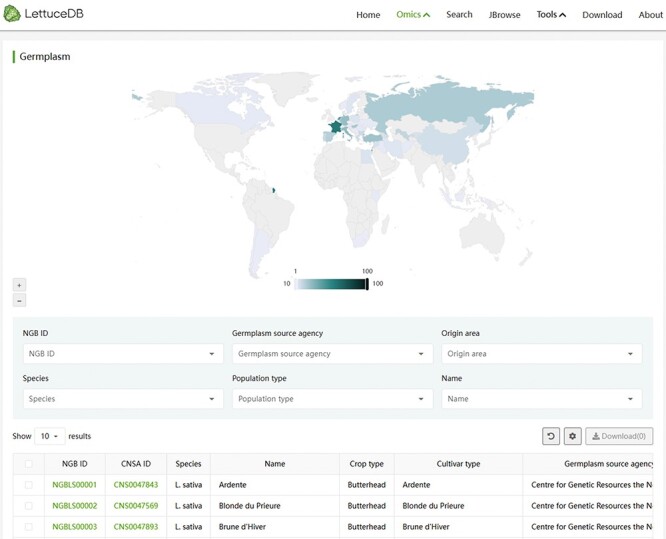
Webpage of the Germplasm module, including an interactive world map of germplasm collection sites and a searching bar for passport information.

The germplasm module provides the passport information for 445 *Lactuca* accessions published in the previous study (15). An interactive world map was inserted showing the global distribution of the investigated accessions (Figure 4). Users can interact with the map by hovering to intuitively understand the distribution of germplasm in each country. At the bottom lies the search field, where users can search for interested germplasm by single or multiple terms, such as National GeneBank (NGB) IDs, germplasm source agencies, origin areas, species names, population types and names. By clicking on the NGB accession number of each germplasm, users can navigate to the detailed information page with the passport information and phenotypic records. Clicking on the China National GeneBank (CNGB) Sequence Archive links will redirect the detailed information page of the sample information and sequencing data. Overall, the germplasm module provides a convenient way to browse and query the lettuce germplasm resources and gain the collective information of each accession.

### The genome module

**Figure 5.**
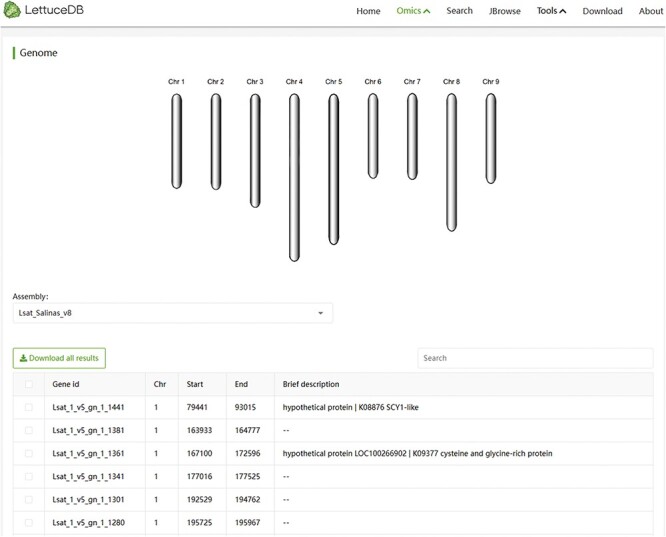
Webpage of the Genome module, including a diagram of chromosome structure and a searching bar for annotation information.

The Genome module offers two versions, Versions 8 and 11, of gene annotations for cultivated lettuce, which can be selected from a dropdown menu (Figure 5). The page includes a lettuce chromosome structure diagram and a gene information table browser. The chromosome structure diagram shows the number and size of each chromosome. The search bar, which is above the table browser, allows users to search the gene annotation. The table browser provides gene IDs, coordinates and brief descriptions based on the search result. The Genome module offers an interface for browsing and querying genes and provides annotation of individual genes across different versions.

### The Variome module

**Figure 6.**
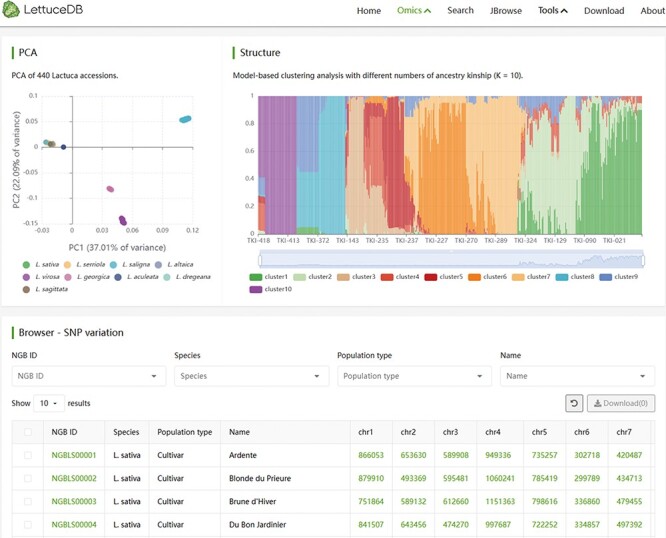
Webpage of the Variome module, including interactive plots of PCA and structure, and a searching bar for SNP counts in each line.

The Variome module encompasses two filtered SNP sets based on two versions of genome assembly. The module page shows population analysis results of the 445 *Lactuca* accessions based on two SNP sets (Figure 6). Users can investigate the genetic relationship and population structure of the germplasm through interactive PCA and population structure plots. These plots are scalable and display germplasm names and classification information. Users can search for specific germplasm variation information based on criteria such as NGB IDs, species, population types and names. The table browser presents NGB accession IDs, species, population types, names and variation site counts for each germplasm. By clicking on the accession number, users can access detailed information from the germplasm page, while the variation count provides a link to JBrowse. In summary, the Variome module offers a comprehensive collection of variation data, allowing for easy exploration of the population structure among different germplasm.

### The Phenome module

**Figure 7.**
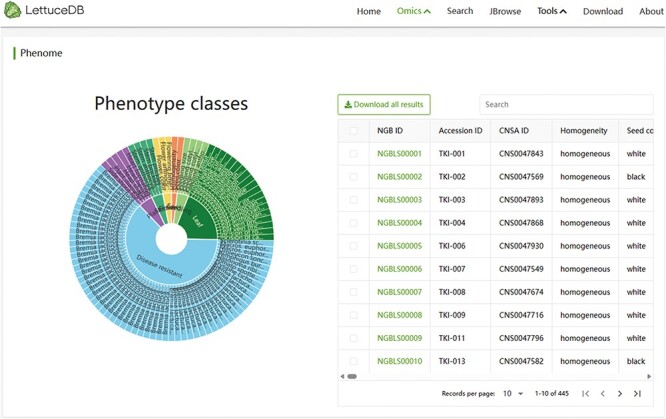
Webpage of the Phenome module, including a sunburst chart of agronomic traits and a searching bar for phenotypic traits.

The Phenome module includes 5419 records of 37 agronomic traits, categorized into seed, seeding, plant, stem, leaf and flower traits, and 62 disease resistances. Interactive visualization of traits is provided through a sunburst chart (Figure 7). Phenotypes can be browsed and filtered by clicking on specific categories or traits in the sunburst chart. The filtered phenotypic information is synchronously shown in the table browser. Specific phenotype information can also be found using the search bar above the table browser. Detailed germplasm information pages can be accessed by clicking on an NGB accession number. In summary, The Phenome module provides researchers and breeders with valuable phenotypic resources, offering flexible retrieval methods and intuitive taxonomic displays.

### The Microbiome module

**Figure 8.**
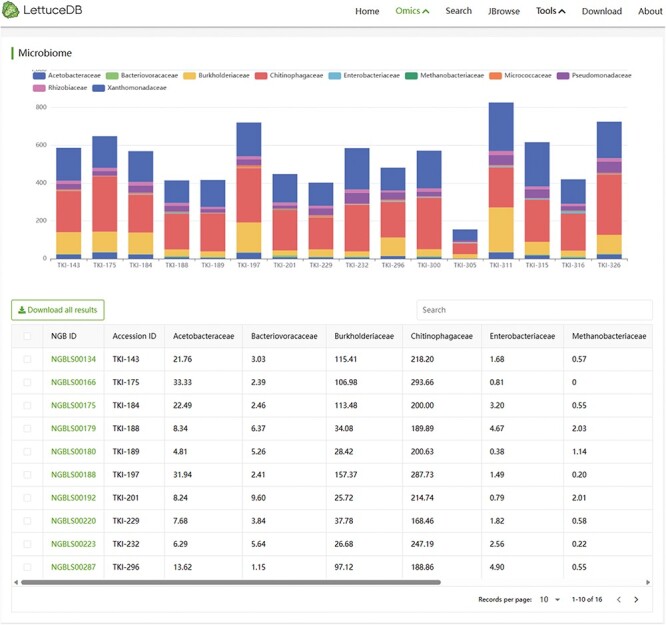
Webpage of the Microbiome module, including a bar chart and a searching bar for microbial abundance.

The Microbiome module provides the abundance of various microbial species in different samples, which can be visually examined through bar charts (Figure 8). A table browser is provided for searching specific species categories and their corresponding abundance information in each sample using keywords. The details page of a species can be accessed by clicking on its ID number. The Microbiome module offers an intuitive and convenient insight into the rhizosphere microbiota associated with lettuce germplasm.

### The Spatial Omics module

**Figure 9.**
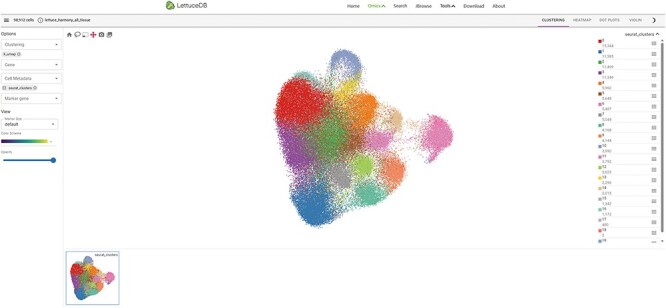
Webpage of the Spatial Omics module, including the main canvas in the center, toolbar on the top, section selector on left, sidebar on right and gallery at the bottom.

The Spatial Omics transcriptome collects single-cell and spatial transcriptomics data of lettuce leaves. The module page is divided into five sections: a section selector, a top toolbar, a sidebar, a main canvas and a gallery (Figure 9). The section selector at the top enables users to choose a specific section within the dataset. On the left side of the top toolbar, the number of cells and the number of selected cells in the dataset are displayed. On the right side, there are three buttons: EMBEDDINGS, DISTRIBUTIONS and a moon symbol. The EMBEDDINGS button displays the default canvas interface. The DISTRIBUTIONS option allows users to explore the differential gene expression across cell clusters with a dot plot, a heat map or a violin plot. The moon symbol button is the dark model option. The left sidebar provides options for selecting genes/traits to visualize, choosing clustering tags and performing differential expression analysis. The main canvas shows an interactive 2D or 3D graphic, which can be panned, zoomed and selected in specific regions using the mouse. The gallery, which is located in the bottom, shows thumbnails of selected genes/gene sets. Users can click on a thumbnail to display it on the main canvas.

### Application toolkits

**Figure 10.**
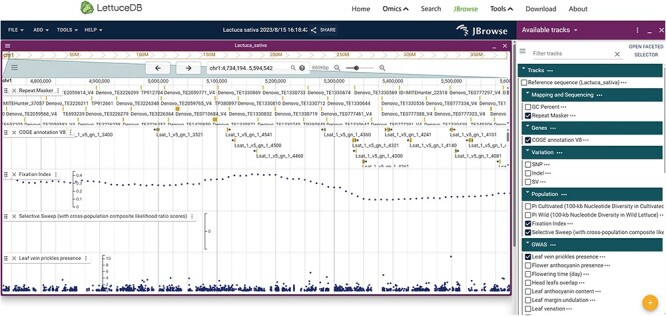
Webpage of the Jbrowse, including gene annotation, sequence variations, selection regions and genome-wide association results.

**Figure 11.**
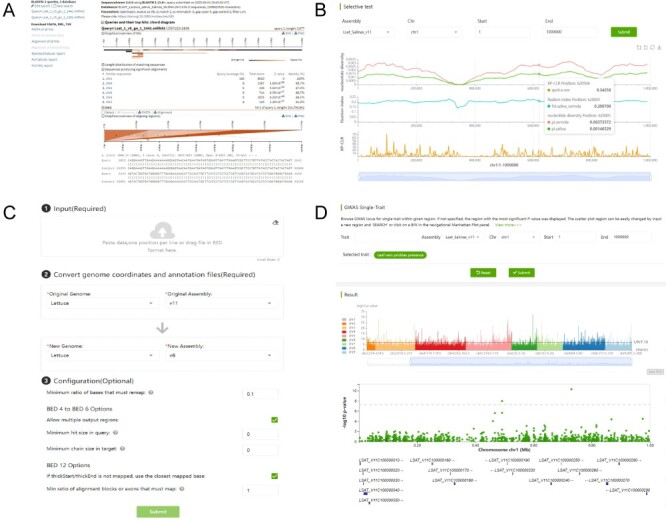
Webpage of the bioinformatics tools, including BLAST (A), selective test (B), LiftOver (C) and GWAS Single-Trait (D).

In addition to the six modules, we incorporated commonly used JBrowse, BLAST, LiftOver, Selective test and GWAS Single-Trait to facilitate laboratory research (Figures 10, 11). In the genome browser JBrowse, gene functions, genetic variations and associations signals were shown for an intuitive understanding (Figure 10). Five types of tracks can be navigated in the browser: the first encompasses reference sequence, GC percent and repeat-masked regions; the second presents gene annotation; the third comprises sequence variants including SNPs, InDels and Structure Variation; the fourth presents population statistics, including nucleotide diversity ($\pi $), fixation index (*F*_ST_) and selective sweeps between cultivated and wild lettuce; the fifth provides genome-wide association results for 30 agronomic traits. JBrowse allows users to navigate all data tracks associated with the lettuce reference genome.

The LiftOver is provided for converting genome coordinates between genome assemblies, and currently, it supports the conversion between the v8 and v11 assemblies (Figure 11C). The selective test provides $\pi $, ${F_{{\mathrm{ST}}}}$ and selective sweeps based on two versions of genome assemblies, from which users can identify genomic regions under selection with reduced diversity in cultivated lettuce and high XP-CLR scores (Figure 11B). GWAS Single-Trait can be used to query the association result for each trait, by which desired traits and regions can be queried using the input boxes at the top. Regions are conveniently explored by clicking on the histogram within the interactive Manhattan plot track, and a zoom-in view illustrates the significance for each variant within the selected genomic region (Figure 11D).

### A case study using the omics data in LettuceDB

**Figure 12.**
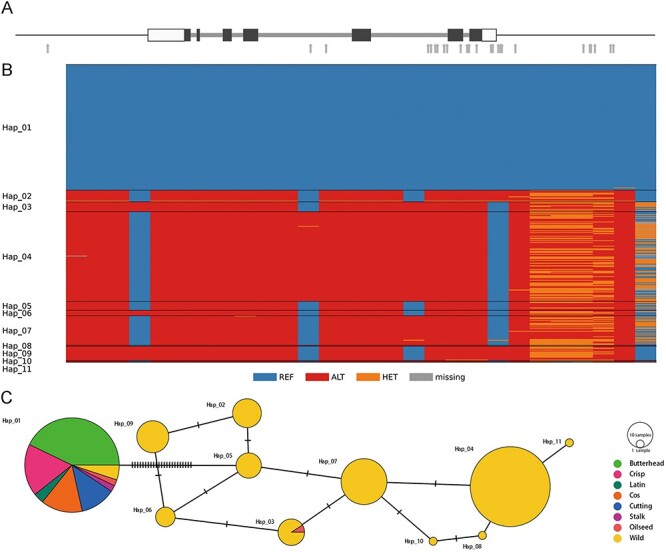
Genetic diversity in the lettuce KN1 gene. (A) Gene structure of Lsat_1_v5_gn_7_15020 that encodes the KN1 transcription factor. Black bars denote coding regions, and white bars represent 5ʹ- and 3ʹ-untranslated regions. Gray arrows indicate SNPs in their genic and flanking regions. (B) Genotypes of 28 SNPs in the genic and flanking regions in 332 cultivated and wild accessions (C) A median-joining haplotype network.

To demonstrate the usage of multi-omics data, we searched the LettuceDB with a previously published *KN1* gene involved in leaf development (21). We searched the lettuce reference genome using the *KN1* coding sequence by BLASTN and found the corresponding *Lsat_1_v5_gn_7_15020* containing 16 SNPs in its genic region, one in the 2-kb upstream and 11 in the 2-kb downstream region (Figure 12A and B). To investigate the genetic diversity of *KN1* in cultivated and wild lettuce, we constructed a haplotype network using the 28 SNPs from 332 *L. sativa* and *L. serriola* accessions (Figure 12C). We found that the majority of cultivated lettuce possessed the same haplotype, *KN1^Hap01^*, along with seven *L. serriola* accessions, which were originated from Iraq, Israel and Romania. This result indicates the *KN1* allele prevalent in modern cultivars was most likely inherited from ancestral wild accessions close to the domestication center (15).

## Discussion

LettuceDB was developed in this study to integrate multi-omics data and bioinformatics tools for cultivated lettuce. It effectively aligns with the current trend of accumulating omics databases in the post-genomics era, particularly in terms of multi-omics interaction and online analysis functionality.

Despite the richness of data type in LettuceDB, concerted efforts are still needed to enhance data sharing and integration. For instance, multi-omics data from 1333 germplasm have been integrated into the Lettuce Genome Database (LettuceGDB) (https://lettucegdb.com/), representing additional data resource (22). However, because different collections of germplasm were used, it is difficult to directly compare the results between databases. Therefore, collaborative partnerships and follow-up meta-analyses are required to expand the range of data sources. In rice, a new variome was recently built from the public resequencing data of 10 548 germplasm, resulting in more than 65 million variants, from which a comprehensive variation database, Rice Super-Population Variation Map, was built (23). Integration of multi-omics data from various sources (24) would facilitate a more comprehensive data mining attempt and ensure a more holistic perspective.

Moving forward, the future direction of LettuceDB primarily revolves around continuous integration of newly generated omics data and development of user-friendly bioinformatics tools for both data scientists and laboratory researchers. Leveraging artificial intelligence–based approaches for deep mining of these datasets holds a great promise in providing valuable insights. For instance, more than two thousand papers collected by LettuceGDB provide a valuable literature source (22), on which using the latest generative language model would potentially provide unprecedented knowledge for the research society.

In summary, the present LettuceDB provides a one-stop platform for muti-omics data sharing and integrative data analysis, which would facilitate lettuce research and breeding in the future.

## Data Availability

All data described in this manuscript are available at https://db.cngb.org/lettuce/.
